# Chronic Obstructive Pulmonary Disease (COPD) management in the community: how could primary care team contribute?

**DOI:** 10.1186/s12875-020-01256-0

**Published:** 2020-09-08

**Authors:** X. R. Catherine Chen, S. H. Leung, Y. C. Li

**Affiliations:** Department of Family Medicine and General Outpatient Clinics (GOPCs), Kowloon Central Cluster (KCC), Hospital Authority (HA), Kowloon, Hong Kong

**Keywords:** COPD, Clinical audit, Primary care, Quality improvement

## Abstract

**Background:**

Chronic obstructive pulmonary disease (COPD) is a progressive lung disease commonly encountered in primary care. This study aimed to audit COPD care at primary care clinics of Hong Kong and to work out improvement strategies.

**Method:**

All COPD patients aged 40 or above who had been followed up at 13 public primary care clinics of Kowloon Central Cluster (KCC) under the Hospital Authority of Hong Kong (HAHK) were included in this clinic audit. Evidence-based audit criteria and performance standards were established after thorough literature review. Phase 1 was from 1st April 2016 to 31st March 2017, with deficiencies of care identified. It was followed by a one-year implementation phase through which a series of improvement strategies were executed. Outcome of the enhancement was reviewed during Phase 2 from 1st April 2018 to 31st March 2019. Chi-square test and student’s t test were used to detect statistically significant changes between Phase 1 and Phase 2.

**Results:**

A total of 2358 COPD cases were identified in Phase 1 where 658 of them were smokers. Of those smokers, 332 (50.5%) had been referred to Smoking Counselling and Cessation Service (SCCS) and 289 (43.9%) actually attended it. 991 cases (42%) received Seasonal Influenza Vaccine (SIV) and 938 cases (39.8%) received Pneumococcal Vaccine (PCV). 698 patients (29.6%) had spirometry done before and 423 patients (17.9%) had been admitted to hospital due to acute exacerbation of COPD (AECOPD). With the concerted effort taken during the implementation phase, Phase 2 data showed significant improvement in nearly all criteria. There was a marked increase in the SIV and PCV uptake rate, spirometry performance rate and most importantly, a significant reduction in AECOPD rate leading to hospital admission (13.5%, *P* = 0.000043). However, the referral rate and attendance rate of SCCS among smokers remained stagnant (*P* > 0.05).

**Conclusion:**

Via a systematic team approach, COPD care at primary care clinics of KCC under HAHK had been significantly improved for most of the audit criteria, which in turn reduced the burden of the healthcare system.

## Background

Chronic obstructive pulmonary disease (COPD) is a progressive lung disease commonly encountered in primary care. According to The Global Burden of Disease Study, there were 251 million COPD cases in 2016 around the world and around  3.17 million people died from COPD in 2015 [1]. In Hong Kong, the prevalence of COPD was about 3.5% in 2000 according to a population based survey [2]. Another local study estimated the prevalence rate of COPD among people aged 60 and above was 25.9% based on lung function test [3]. Furthermore, COPD accounted for almost 30,000 hospital admissions in the Hospital Authority of Hong Kong (HAHK) and resulted in 2.7% of all registered death in 2016 [4]. Therefore, COPD represents a substantial economic and social burden to the health care system.

The chronic disease nature of COPD warrants regular patient follow-up and multi-disciplinary team assessment to optimize its control. As cigarette smoking is a major cause of COPD, smoking cessation is the most effective intervention to slow down its disease progression. Therefore, all professionals should, at every opportunity, advise and encourage smokers to stop smoking. Other aspects of COPD management such as proper diagnosis by spirometry and implementation of influenza and pneumococcal vaccination have all been shown to reduce the disease burden and improve patients’ quality of life [5–7]. Despite all these evidences, the management of COPD is still far from satisfactory. For example, smoking cessation can be very difficult among COPD smokers, and they have a particularly low referral rate and attendance rate for smoking cessation counselling service [8, 9]. Furthermore, Kester et al. found that only 5% of Canadian general practitioners requested a pulmonary function test when attending an individual with clear signs of COPD [10]. It is also not surprising that the actual take-up rate of seasonal influenza vaccine has consistently been low at 20–60% [11–14]. If all these preventive measures are not effectively implemented, acute exacerbation of COPD (AECOPD) would be inevitable. Indeed, acute exacerbations have been proven to lead to accelerated decline in lung function, poorer overall health status, COPD-related hospitalizations and mortality, and lastly increased the health care utilization [15].

About 80% of COPD patients are managed under specialist care and 20% are managed under primary care within HAHK [16]. A pilot survey conducted in 2011 found that COPD care at both primary and secondary level in HAHK needed to be improved [17]. Furthermore, a suboptimal adherence to accredited COPD management guideline was identified at five tertiary respiratory centers from 2013 to 2015 [18]. Insufficient patient education, under-treatment of COPD and lack of an integrative management model were the main issues to be tackled. In view of this, starting from 1 April, 2017, COPD audit had been conducted across all primary care clinics of HAHK to review the performance of COPD care so as to improve its clinical outcome. The goal of this study was to audit the management of COPD cases from 13 primary care clinics of HAHK and to work out improvement strategies. Primary care professionals have an important role in improving the quality of care of COPD patients. We believe that by improving the standard of care to COPD patients managed in the community, the disease burden including the number of hospital admissions due to AECOPD would be greatly reduced.

## Method

### Study design

A two-phase clinic audit conducted at 13 public primary care clinics of Kowloon Central Cluster (KCC) of the HAHK.

### Setting the audit criteria and performance standards

Guidelines on COPD management published in recent 3 years were identified from the PubMed (https://pubmed.ncbi.nlm.nih.gov/). After thorough literature review, members of Quality Assurance Subcommittee of Coordination Committee of Family Medicine of HAHK had recommended adopting the following evidence-based audit criteria and performance standards for the COPD audit across all primary care clinics in HAHK as summarized in Table 1. They were mainly based on the recommendations from the following two accredited guidelines on COPD manaagement: 

1. **G**lobal Initiative for **O**bstructive **L**ung **D**isease: Global strategy for the diagnosis, management and prevention of chronic obstructive pulmonary disease (GOLD guideline) (2018 report) [19].

2. NICE guideline [NG115] Chronic obstructive pulmonary disease in over 16 s: diagnosis and management, 2018 [20].

### Data collection and analysis

#### Audit objects

Eligible study subjects were COPD patients aged 40 or above and had attended any of the 13 General Outpatient Clinics (GOPCs) of KCC under the HAHK for regular follow-up (FU) during the study period. Totally there are 7 clusters in HAHK, with KCC being the largest hospital cluster with a catchment of 1.2 million populations in 2017. There are 13 GOPCs under the jurisdiction of KCC, and around 15% of the population were aged over 65 years in 2017. The diagnosis of COPD was identified through all GOPC attendances coded with International Classification of Primary Care (Second edition) of ‘R79-Chronic bronchitis’ or ‘R95-Chronic obstructive pulmonary disease’ over a 12-month reporting period. COPD patients managed in the Specialist Outpatient Clinics (SOPCs) or certified dead during the study period were excluded from the data analysis.

### The audit cycle

Phase 1 was from 1st April 2016 to 31st March 2017, with deficiencies of care identified. It was followed by a one-year implementation phase through which a series of improvement strategies were executed. Outcome of the enhancement was reviewed during Phase 2 from 1st April 2018 to 31st March 2019.

#### First-phase data collection and analysis

A total of 2496 COPD patients were found to have regular FUs in 13 GOPCs of KCC during Phase 1. Among them, 125 cases were certified dead and 13 cases were found to have FU at SOPCs and therefore were excluded. The remaining 2358 cases (94.5%) fulfilling the inclusion criteria were included into data analysis.

#### Implementing changes and intervention

At the department level, the COPD audit working group led by the authors was formed on 1st April 2017 and subsequently, doctor and nurse subject officers were assigned. A structured team approach was adopted to set out strategies on administrative policy and management guidelines. COPD case registry had been retrieved from the Clinical Data Analysis and Reporting System of HAHK at least annually in which the information was shared with clinic doctors and nurses in-charge for follow up actions. Regular service review meeting had been conducted quarterly to half yearly.

At the practice level, the management guideline of COPD cases across all 13 GOPCs were aligned. All COPD cases would be referred to attend a nurse-led program called Nurse and Allied Health Clinic -Respiratory Team upon their routine FU. A comprehensive range of services would be provided including nursing assessment and counselling, spirometry assessment, smoking cessation service referral if smoker, and a course of pulmonary rehabilitation by allied health workers including physiotherapist and occupational therapist. For those COPD cases in the registry but did not have a scheduled appointment, a doctor medical consultation within 6 months would be offered. All COPD cases would receive standard assessment including the Modified Medical Research Council (mMRC) dyspnea scale and COPD Assessment Test (CAT) score evaluation, and then were graded according to the definition of GOLD guideline. Moreover, five more Spirobank machines were purchased to meet the increasing service demand on spirometry assessment.

At the clinic level, a policy on COPD risk factor screening was advocated, and a continuous monitoring-and-feedback system with ongoing problem solving was reinforced. At the doctor level, diagnosis and management of COPD and prevention of acute exacerbation based on the latest GOLD guideline had been promulgated to all frontline doctors to sharpen their skill set. All doctors were advised to manage COPD cases according to their severity as suggested by the grading system, and to make appropriate referrals if deemed necessary. Regular quarterly review on the progress of the audit was carried out where feedback regarding the deficiencies was tackled promptly. At patient level, regular health talk had been organized by various ranks of staff to improve the awareness and knowledge of the patients on COPD care. All COPD smoker patients would be advised to receive the smoking cessation counselling service unless refusal to participate. The deficiencies in service provision and corresponding implementation strategies are summarized in Table 2.

**Table 1:** list of audit criteria and performance standards of the study

Item no.	Recommendations and audit criteria	Standard
1	Recommendations: All COPD patients should be regularly reviewed with a pre-scheduled appointment in General Outpatient Clinic (GOPC); Criteria 1: % of COPD patients with pre-scheduled appointments in GOPC.	85%
2	Recommendations: All smoker COPD patients should be advised on smoking cessation and referred to Smoking Counselling and Cessation Services (SCCS); Criteria 2: COPD patients who are smokers: a. % of COPD patients referred to SCCS before or b. % of COPD patients ever attended SCCS before	50% 45%
3	Recommendations: All COPD patients should receive seasonal influenza vaccine (SIV) annually unless contraindicated; Criteria 3: % of COPD patients who have received SIV in the preceding year.	45%
4	Recommendations: All COPD patients should receive pneumococcal vaccine (PCV) unless contraindicated; Criteria 4: % of COPD patients who have received PCV before.	45%
5	Recommendations: Spirometry is recommended for all COPD cases at diagnosis or when the alternative diagnosis needs to be ruled out, and to monitor the disease progression. Criteria 5: % of COPD patients with spirometry test done before.	50%
6	Recommendations: All efforts should be made to reduce the acute exacerbation pf COPD (AECOPD). Criteria 6: % of COPD patients admitted to hospitals due to AECOPD.	< 15%

**Table 2:** Deficiencies identified and strategies implemented

Areas of deficiencies	Strategies implemented
**Policy**	
Lack of a responsible team	Set up of COPD audit Working Group with members from both doctors and nurse. Appointment of one doctor and one nurse from the working group as the audit coordinator.
Lack of regular review to monitor the COPD management performance	Quarterly review policy to monitor the process
Lack of collaboration with SOPDs	Collaborate with Respiratory Medicine Team, Kowloon Hospital on handling severe COPD cases and download mechanism for stable COPD cases.
**Practice**	
Lack of COPD registry	COPD case registry has been retrieved from CMS and has been updated by the working group quarterly
Lack of guideline or protocol	Adopt standard guidelines, development of protocol and structural COPD assessment form
Lack of aligned workflow	The workflow of managing COPD cases were streamlined across all 13 GOPCs in KCC.
Lack of spirometry machine	Purchased 5 more Spirobank machine to cater for the service demand in local GOPCs.
Lack of drugs for COPD care, such as LAMA.	Newly introduced LAMA to Family Medicine Specialist Clinic in 2018.
**Staff**	
Lack of continuous education and training	Improvement on education and training through workshops, clinical meetings and journal clubs.
Lack of team work	Sharing of workload among staffs of all ranks, including doctors, nurses, clerks, allied health workers such as physiotherapist, occupational therapist and dietitian.
Standardized COPD management workflow	All COPD patients were managed according to the grouping based on the latest GOLD guideline.
Lack of feedback	Quarterly to biannually review on the progress of the audit, deficiencies are tackled promptly.
**Patient**	
Lack of awareness and knowledge and/or lack of motivation on smoking cessation	Improve patient’s awareness and knowledge by regular health talks and nurse counselling.

**Table 3:** Demographic characteristics of COPD patients in the two phases. Data are shown as N (%) of cases or mean +/- standard deviation

	Phase 1	Phase 2	P value
COPD cases included in data analysis (n)	2358	2177	/
Gender			0.80
Male	1981 (84.0%)	1835 (84.3%)	
Female	377 (16.0%)	342 (15.7%)	
Age (years)	75.9 ± 10.6	75.8 ± 10.7	0.82
< 65 years old	368 (15.6%)	339 (15.6%)	0.97
≥65 years old	1990 (84.4%)	1838 (84.4%)	
Smoker	658 (27.9%)	661 (30.4%)	0.07
Body Mass Index (BMI, kg/m^2^)	23.1 ± 4.0	23.4 ± 4.1	0.76
Underweight (BMI < 18.5)	314 (13.3%)	263 (12.1%)	0.06
Normal (BMI 18.5–22.9)	915 (38.8%)	797 (36.6%)	
Overweight (BMI 23.0–24.9)	405 (17.2%)	433 (19.9%)	
Obesity (BMI > 25)	724 (30.7%)	684 (31.4%)

**Table 4:** Severity of COPD cases in Phase 1 and Phase 2 according to GOLD guideline. Data are shown as N (%)

GOLD guideline group	Phase 1 (***n*** = 2358)	Phase 2 (***n*** = 2177)	P value
Group A	1820 (77.2%)	1663 (76.4%)	0.31
Group B	399 (16.9%)	359 (16.5%)	
Group C	78 (3.3%)	79 (3.6%)	
Group D	61 (2.6%)	76 (3.5%)	

Group A: 0 or 1 moderate exacerbations (not leading to hospital admissions) AND mMRC 0–1 or CAT< 10.

Group B: 0 or 1 moderate exacerbations (not leading to hospital admissions) AND mMRC ≥2 or CAT ≥10.

Group C: ≥2 moderate exacerbations or ≥ 1 leading to hospitalization AND mMRC 0–1 or CAT< 10.

Group D: ≥2 moderate exacerbations or ≥ 1 leading to hospitalization AND mMRC ≥2 or CAT ≥10.

CAT = COPD assessment test; mMRC = modified Medical Research Council dyspnoea questionnaire.

**Table 5:** Number and percentage of patients with criteria fulfilled in Phase 1 and Phase 2 and comparison of the results in the two phases

Item no.	Audit criteria	Phase 1 (n = 2358)	Phase 2 (n = 2177)	P value
1	COPD patients with pre-scheduled appointments in General Outpatient Clinic (GOPC)	1886 (80.0%)	1997 (87.6%)	< 0.00001
2	COPD patients who are smokers	658 (27.9%)	*n* = 661 (30.4%)	0.069
	a. have ever been referred to Smoking Counselling and Cessation Services (SCCS) before or	332 (50.5%)	367 (55.5%)	0.065
	b. have ever attended SCCS before	289 (43.9%)	317 (48.0%)	0.141
3	COPD patients who have received seasonal influenza vaccine (SIV) in the preceding year;	991 (42.0%)	1072 (49.2%)	< 0.00001
	< 65 years old	28/368 (7.6%)	78/339 (23.0%)	< 0.00001
	>= 65 years old	963/1990 (48.4%)	994/1838 (54.1%)	< 0.00001
4	COPD patients with have received pneumococcal vaccine (PCV) before;	938 (39.8%)	1244 (57.1%)	< 0.00001
5	COPD patients with spirometry test done before	698 (29.6%)	1582 (72.7%)	< 0.00001
6	COPD patients admitted to hospitals due to acute exacerbation (AECOPD).	423 (17.9%)	294 (13.5%)	0.000043

Data are shown as No. (%)

#### Second-phase data collection and analysis

A total of 2282 COPD patients were found to have regular FU in GOPCs of KCC during Phase 2. Of those being excluded in this phase, 103 cases were certified dead and 2 cases were found to have FU at SOPDs. The remaining 2177 cases (95.4%) were included into data analysis.

### Determination of variables

The recruited patients’ age, gender, smoking status, body mass index (BMI) were retrieved.

from the Clinical Management System (CMS) of the HAHK. The BMI was calculated as body weight/body height^2^ (kg/m^2^). The severity of COPD symptoms was graded from Group A to D according to the GOLD guideline [19]. All data of the 6 audit criteria were retrieved from the Clinical Data Analysis and Reporting System of HAHK by head office statistics team.

### Statistical methods

All data were entered and analyzed using computer software (SPSS version 16.0; Inc., Chicago [IL], US). Chi-square test and independent student’s t test were used respectively for categorical and continuous variables to examine the statistically significant differences between the first phase and second phase measurements. A *P* value of < 0.05 (two-tailed) was regarded as statistically significant.

## Results

Table 3 summarizes the demographic characteristics of COPD patients included into the two phases. Among the 2358 patients reviewed in Phase 1, 270 cases (11.5%) were referred to specialists for continued care. Among the 2177 patients included in Phase 2, 1578 patients were FU cases from Phase 1, with a case overlapping rate of 72.5%. The demographic profiles of patients in the two phases were comparable, with around 84% being male and the majority were elderly patients aged over 65 years old. Approximately 30% of COPD patient were chronic smokers and 12–13% of them were underweight. Further review on the severity of COPD cases included in the two phases revealed that their grading of GOLD Group A-D was similar too (Table 4). More than 90% of COPD cases belonged to the relatively stable Group of A or B.

A comparison of the standards achieved in the two phases is summarized in Table 5. In the first phase, marked deficiencies were identified in almost all criteria. It is quite alarming to note that less than one third of COPD patients (29.6%) had performed spirometry before and 423 cases (17.9%) had at least one episode of hospital admission due to AECOPD. After proactive execution of the enhancement strategies during the implementation phase, significant improvement was observed with respect to most of these criteria in Phase 2. The improvement was impressive for arrangement of regular pre-scheduled FUs, seasonal influenza vaccination rate and pneumococcal vaccination rate (all *P* < 0.00001). The most clinically important change was observed in the spirometry performance rate and hospital admission rate due to AECOPD. Regretfully, despite the concerted effort on promotion of SCCS to all smoker COPD patients, the referral rate and attendance rate at SCCS had not significantly improved. Regardless, there was an observed improving trend, and target standard had been achieved.

## Discussion

Our study revealed that deficiencies existed in various aspects of COPD management in the primary care setting. These were particularly apparent in the process criteria such as smoking cessation service referral, uptake rate of SIV or PCV, and under-utilization of spirometry test. Through the audit process, COPD management at primary care level had been greatly enhanced, with most audit criteria being markedly improved and target audit standard achieved. The outcome criteria, AECOPD rate leading to hospital admission, was also significantly reduced in Phase 2.

In Phase 1, loopholes were identified in various aspects of COPD management. About 20% of COPD patients were found not to have a prescheduled FU for regular assessment at the beginning of the audit. FU care is essential as it allows further discussion on the management plans and future monitoring. To resolve this issue, the audit team had gone through the CMS record of this group of patients to review whether they had been arranged to have any FUs with private doctors or respiratory specialists. All of them were called up individually by nursing staff to enquire about their symptom control. For those without any proper assessment in the recent 1 year, an appointment for doctor’s consultation in their respective GOPC within 6 months would be offered. With such effort, many lost-to-FU COPD cases returned to GOPC for spirometry and clinical assessment, with 87.6% of all COPD cases having regular FU in GOPCs in Phase 2 (*P* < 0.0001).

A combination of behavioral and pharmaceutical interventions has been provided at the SCCS in our department since 2010. Although we encouraged all smoker COPD patients to attend the SCCS during their routine doctor consultation, only about half of them were referred and even fewer (43.9%) actually attended in Phase 1. Despite our proactive promulgation, the situation during Phase 2 had not significantly improved although a rising trend was observed and the target standard had been achieved. These findings were consistent with literature suggesting that COPD smokers are poorly motivated to quit smoking in general [9]. Another possibility is the physician-related factor. For example, literature shows that some physicians do not routinely deal with smoking cessation during their consultations with smokers partly due to lack of cessation specific knowledge or skills and partly due to insufficient consultation time [21]. This is particularly true in our local GOPCs where the average time allocated for each consultation is only 6 to 7 min. There is definitely a need for a more proactive approach to promote the smoking cessation among all health care workers and COPD patients.

For criteria 3 and 4 on the update rate of SIV and PCV, although our performance had significantly improved during the audit cycle, only about half of COPD patients were vaccinated against SIV (49.2%) and PCV (57.1%) in Phase 2. Indeed, influenza vaccination coverage rates among COPD patients remain low in many countries [12, 13]. In HK, all elderly patients aged over 65 years are entitled to receive SIV and PCV for free under the HK Government Vaccination Program. However, the breakdown figures of SIV coverage among those under age 65 were still far from satisfactory (7.6% in Phase 1 and 23.0% in Phase 2). Given the widely established evidence on the long-term benefits of SIV on COPD care, such as reduced number of exacerbations, hospitalizations and all-cause mortalities [6], we would like to propose that HK government should launch out free SIV to COPD patients of all ages to reduce the mortality.

It is disappointing to find that only 29.6% COPD patients had ever conducted spirometry test in Phase 1. The reasons accounting for this poor performance rate are multifactorial. At doctors’ level, some doctors often make the diagnosis of COPD based on clinical features alone. At clinic level, spirometry service was previously only available at hospital setting. Therefore, all suspected COPD patients had to be referred to Respiratory Specialist Clinic to perform lung function tests where the waiting time ranged from months to 2 years under HAHK. To plug this loophole, a series of education talks on the proper diagnosis and management of COPD, emphasizing on the importance of spirometry test, were delivered to all doctors. Furthermore, almost all GOPCs were equipped with spirometry machine during the implementation phase so that the spirometry test could be conveniently performed locally within 2–4 weeks. In addition, at least 1–2 designated nurses from each GOPC had been specially trained on how to perform the spirometry correctly based on the aligned standard. With such facilitations both on the skill set and tool set, it is not surprising that tremendous improvement was observed for this criteria in Phase 2 (72.7%, *P* < 0.00001).

The last criteria 6, the rate of AECOPD leading to hospital admission, is the single most important outcome criteria of this audit. Mild to moderate AECOPD that had been well managed in GOPCs would not be included in this criteria. In Phase 1, we found it quite alarming that almost 1 in 5 of COPD patient (17.9%) had been admitted to hospital due to AECOPD during the audit year. This data was comparable to Canadian studies which showed that approximately 20% of COPD patients had experienced severe acute exacerbations annually [22]. In order to decrease the burden of hospital admissions, prevention and prompt treatment of exacerbations are the key goals in COPD care. In view of this, a series of service enhancement strategies were executed. Firstly, early identification of COPD patients by spirometry and proper grading according to the GOLD guideline were done as mentioned above. Secondly, all COPD cases were managed according to their grading, hence providing the right level of care to the right patients. For example, stable Group A patients would continue regular FU at GOPCs, where only a limited pharmacological choices including short-acting bronchodilators are available. Group B patients would be managed at Family Medicine Specialist Clinics, where long acting antimuscarinic antagonist (LAMA) was newly introduced in 2018 to improve their symptom control. A more comprehensive assessment would also be provided by the experienced Family Medicine Specialists at the clinic. For more severe Group C or D patients that warrant advanced care, a timely referral to the respiratory specialists would be initiated. Lastly, relatively stable AECOPD patients could be successfully managed at outpatient settings with more frequent FU instead of being admitted to hospital, hence reducing the hospital burden. This is in line with findings in the literature which state that frequent outpatient visits prevent exacerbation of COPD [23]. With all these proactive interventions and efforts, the AECOPD rate leading to hospital admission was significantly reduced to 13.5% in Phase 2.

### Strength and limitations of this study

COPD is one of the most important disease entity commonly encountered in primary care. Moreover, COPD patients are one of the most vulnerable group of population in the community. Therefore, clinical audit on this topic and the subsequent continuous quality improvement programs will likely to have tremendous impact on COPD care in the community. To our knowledge, this study is one of the biggest clinical audits on COPD management ever conducted both locally and internationally, and it has provided crucial information on COPD care in primary care setting. The sample size was quite large with more than 2000 cases included in both Phase 1 and 2. The broad spectrum of audit criteria evaluated in the study reflected the comprehensive nature of COPD management in primary care. In addition, all audit criteria were based on objective assessment parameters with data being retrieved from the computer system from HAHK, therefore minimizing recall basis or data entry error.

With that being said, this study has several limitations. Firstly, the study was carried out in one single cluster of HAHK, therefore selection bias might be present. These results from the public primary health care sector might not be applicable to the private sector or secondary care. Nevertheless, since COPD cases from all 13 GOPCs of KCC had participated in the clinic audit, these data may give a realistic representation of COPD care in the public primary care settings and had provided important background information for future service enhancement. Secondly, this clinical audit mainly focused on short-term outcome aspects of COPD management. Long-term outcomes such as lung function improvement, smoking cessation rate, or COPD-related mortality rate were not analyzed. In addition, some process indicators such as assessment on inhaler technique and drug adherence had not been included. This gap will need to be filled as evidence has revealed that inhaler technique education significantly reduced exacerbation rate [24], and is shown to be more cost-effective [25]. Subsequent studies focusing on the long-term outcome criteria and inhaler technique may help provide a more comprehensive picture of COPD management. Lastly, the one-year intervention phase might not be long enough for some criteria to achieve the statistically significant change, although clear improvements were shown. Therefore, continuous effort to implement more audit cycles would be necessary to further improve the clinic outcome in COPD care.

## Conclusion

COPD management at primary care clinics of KCC under HAHK had been significantly enhanced during the past 2 years. By applying a team approach with streamlined governance and structure as well as proactive staff engagement, marked improvement had been achieved in most of the audit criteria for COPD management. These augmentation strategies had significantly reduced the hospital admission rate of COPD patients due to acute exacerbation, subsequently reduced the burden to specialist care and hospital.

## Data Availability

The datasets used in the current study were compiled by the hospital statistical team from the head office of HAHK and are used as internal reference only. They would be available from the corresponding author on reasonable request after being approved by headquarters of HAHK.

## Abbreviations

AECOPD: Acute Exacerbation of Chronic Obstructive Pulmonary Decease
BMI: Body Mass Index
CAT: COPD Assessment Test
CMS: Clinical Management System
COPD: Chronic Obstructive Pulmonary Disease
FU: Follow Up
GOLD guideline: Global Initiative for Obstructive Lung Disease guideline
GOPCs: General Outpatient Clinics
HAHK: Hospital Authority of Hong Kong
KCC: Kowloon Central Cluster
LAMA: Long Acting Anti-muscarinic Antagonist
mMRC: Modified Medical Research Council
PCV: Pneumococcal Vaccine
SCCS: Smoking Counselling and Cessation Service
SIV: Seasonal Influenza Vaccine
SOPCs: Specialist Outpatient Clinics
